# From Algorithm to Care: AI-Assisted Glaucoma Screening in Real-World Settings

**DOI:** 10.7759/cureus.117894

**Published:** 2026-10-14

**Authors:** Hemlata Udenia, Vivek Gupta, Praveen Vashist, Tanuj Dada

**Affiliations:** 1 Ophthalmology, Dr Rajendra Prasad Centre for Ophthalmic Sciences, All India Institute of Medical Sciences, New Delhi, IND

**Keywords:** artificial intelligence, community eye care, fundus photography, glaucoma screening, portable perimetry, primary eye care, referral pathways

## Abstract

Glaucoma screening is increasingly being discussed in the context of artificial intelligence (AI), particularly as imaging technologies become more accessible outside specialist clinics. The question, however, is not simply whether AI can identify a suspicious optic nerve head, but whether it can contribute to a screening pathway that works in real-world clinical settings. Fundus image-based AI has shown promising performance and is now being explored in community and primary eye care. The clinical value of these advances, however, depends on how effectively AI-assisted screening integrates into routine care pathways. Media opacity and poor-quality images can limit assessment, indiscriminate screening can generate unnecessary referrals, and many patients who are identified as requiring further evaluation may not complete the referral pathway. In glaucoma, the value of AI-assisted screening depends on its ability to facilitate appropriate clinical assessment and subsequent care for those identified as being at risk. AI is therefore best considered one part of a screening strategy rather than a replacement for clinical assessment. Risk-based selection, early recognition of ungradable images, and appropriate management of visually significant cataracts or other media opacity may make screening more efficient. Portable or tablet-based visual field testing could add functional assessment at the primary-care level, although its role remains to be established. The next phase of AI-based glaucoma screening should therefore focus less on improving performance metrics in isolation and more on understanding where the technology fits within existing eye-care services. The ultimate measure of success is not the number of suspicious optic nerve heads an algorithm identifies, but whether people with previously undetected glaucoma reach timely diagnosis, treatment, and ongoing care.

## Introduction and background

Glaucoma is a growing public health challenge, and many cases remain undiagnosed until irreversible vision loss has occurred [1]. A recent synthesis of population-based studies estimated a glaucoma prevalence in India of 3.23% among adults aged 40 years and older, projected to rise to 4.52% by 2050 [2]. However, conventional diagnosis relies on a combination of clinical examination, assessment of the optic nerve head and intraocular pressure (IOP), structural imaging with optical coherence tomography (OCT), and functional assessment with visual field testing. This resource-intensive approach is difficult to scale for population-level screening.

Artificial intelligence (AI), particularly deep learning (DL) applied to fundus photographs, has therefore attracted considerable interest as a potential solution to this scalability gap. Early studies showed that AI could identify glaucomatous optic neuropathy (GON) from color fundus photographs with high diagnostic accuracy. Li et al. showed that a DL system could detect GON across multiple datasets, while Hemelings et al. showed that DL models could extract diagnostically relevant information from retinal regions beyond the optic disc [3,4]. Together, these studies established proof of principle that a single retinal photograph may contain sufficient information for AI to detect glaucoma-related patterns.

However, developing an accurate algorithm is only the first step. A model developed and validated on carefully selected images does not automatically become a screening service. In practice, someone must acquire the image, often outside a glaucoma clinic; the camera must be practical; the patient may not be dilated; the image must be interpretable; and, most importantly, the result must fit into an existing clinical pathway. In this context, “referable glaucoma” refers to an individual identified through screening as having findings that warrant further ophthalmic assessment because of suspected GON or other features associated with risk of glaucoma. The focus is therefore shifting from “How accurately can AI detect glaucoma” to a more practical question: “Can AI-based screening actually work in routine eye care and connect patients to the care they need?” Beyond detection, AI is increasingly being explored for disease quantification, prediction, and monitoring across the continuum of care [5]. However, translating these technological capabilities into population-level screening requires attention to factors beyond algorithmic performance, including image acquisition, patient selection, referral pathways, and access to confirmatory assessment.

Scope and focus of the review

This narrative review examines the emerging evidence on AI-assisted glaucoma screening in real-world settings, with particular emphasis on image acquisition, screening personnel, portability, integration with existing primary and community eye-care pathways, referral, quality assurance, and linkage to confirmatory glaucoma care. The review focuses on the practical question of how AI-assisted screening can be incorporated into existing care pathways in a manner that is clinically appropriate, operationally feasible, and connected to timely confirmatory glaucoma evaluation.

Method of literature search

This targeted narrative review drew on a reference collection the authors maintained over several years and was supplemented by searches of PubMed and Scopus on 20 August 2026. The exact PubMed search string was (glaucoma[Title/Abstract]) AND ((artificial[Title/Abstract] AND intelligence[Title/Abstract]) OR (machine[Title/Abstract] AND learning[Title/Abstract]) OR (deep[Title/Abstract] AND learning[Title/Abstract])) AND (deployment[Title/Abstract] OR real-world[Title/Abstract] OR (real[Title/Abstract] AND world[Title/Abstract]) OR (economic[Title/Abstract] AND analysis[Title/Abstract]) OR (primary[Title/Abstract] AND care[Title/Abstract])) AND (screening[Title/Abstract] OR referral[Title/Abstract]). Reference lists of key publications were also examined to identify relevant studies not captured by the database searches. Studies were considered for inclusion when they provided relevant evidence on AI-based glaucoma detection or screening, including diagnostic performance, fundus photograph-based systems, portable or smartphone-based imaging, implementation in primary or community settings, integration with existing screening pathways, referral processes, quality assurance, or economic evaluation. Systematic and scoping reviews, economic evaluations, professional or consensus guidance, policy documents, and relevant non-AI screening and referral-support studies were considered to provide contextual evidence on implementation and linkage to care. Studies concerned solely with algorithm development or retrospective image-dataset validation were not the focus. Selected tertiary-care validation studies were included where they informed portable imaging, offline assessment, or the interpretation of screening performance. VG performed the database searches, and VG and HU selected articles based on their relevance to the review objectives. Duplicate references were identified by visual inspection and removed manually. Only English-language full-text publications were considered; conference abstracts and preprints were excluded.

Because this was a targeted narrative review, articles were selected based on their relevance to the review objectives rather than through a formal systematic review screening process. Formal PRISMA (Preferred Reporting Items for Systematic Reviews and Meta-Analyses)-style accounting of records identified, screened, and excluded and formal risk-of-bias assessment of individual studies were not done. The literature was synthesized narratively, with emphasis on implementation, including image acquisition, screening personnel, portability, image quality, AI-assisted assessment, referral pathways, confirmatory testing, quality assurance, and linkage to treatment and follow-up.

## Review

From validation to deployment

The emerging evidence matters not because individual studies show high-performing algorithms, but because AI is increasingly being tested in real-world settings (rural vision centers, primary-care clinics, established retinal screening programs, and routine glaucoma services) where screening takes place. This shift from algorithmic validation towards implementation raises practical questions about image acquisition, personnel, connectivity, workflow integration, referral, and linkage to confirmatory care.

Indian experience

The experience from the Indian subcontinent is particularly relevant in this context. Upadhyaya et al. evaluated the offline Medios AI glaucoma software using a handheld smartphone-based fundus camera in six satellite vision centers [6]. Vision technicians captured images using non-mydriatic imaging, enabling screening without continuous internet connectivity or the need for a specialist to be present at the time of image acquisition. Similarly, Rao et al. evaluated an offline AI system integrated with a smartphone-based fundus camera, while Senthil et al. examined its performance across different stages of glaucoma [7,8]. The vision-center study provides evidence on screening delivery outside specialist clinics, whereas the tertiary-care studies provide evidence on diagnostic performance in selected clinical populations. These findings support further evaluation of portable, offline AI systems in community screening. In a related community screening context, Shekhawat et al. demonstrated the feasibility of routine fundus photography for detecting posterior segment disease, providing evidence that retinal imaging can be incorporated into community-based screening workflows [9].

Global experience

Similar implementation-oriented approaches have been evaluated in other healthcare settings. Jan et al. evaluated automated retinal photographs with AI-based glaucoma screening during routine general practice visits in Australia, showing that glaucoma assessment could be incorporated into primary care rather than requiring a dedicated ophthalmic encounter [10]. In Portugal, Lima-Cabrita et al. extended this concept by incorporating AI-based glaucoma screening into an existing diabetic retinopathy screening pathway [11]. Another study from the same group evaluated DL as a tool within the glaucoma referral pathway using simulated model-based referrals [12]. These approaches are important because they make use of existing infrastructure, rather than requiring the health system to develop a separate glaucoma screening program.

The practical differences between these studies may be more informative than their reported performance metrics alone. Table 1 summarizes these real-world implementation studies, distinguishing their populations, settings, screening personnel, imaging arrangements, and reported diagnostic or implementation outcomes.

**Table 1:** Selected studies evaluating AI-assisted glaucoma screening and its implementation in clinical and primary-care settings Abbreviations: AI, artificial intelligence; BCVA, best-corrected visual acuity; IOP, intraocular pressure; OCT, optical coherence tomography; SD-OCT, spectral-domain optical coherence tomography; VFA, visual field analysis; LOCS III, Lens Opacities Classification System III; ICER: incremental cost-effectiveness ratio. “No” indicates that pharmacological pupil dilation was not routinely used for image acquisition; where dilation was used selectively, this is indicated separately.

Study (year)	Settings/evidence type	Population	Screening personnel	Camera	Mydriasis	AI platform	Ancillary investigation	Reported diagnostic or implementation outcomes
Upadhyaya et al. 2025 [6]	6 rural vision centers, Pondicherry, India (community implementation)	Aged 18 years and older	Vision technician	Portable fundus camera	Only if non-mydriatic images were inadequate	Inbuilt offline AI algorithm	BCVA, slit lamp, Goldmann applanation tonometry	299/303 participants had gradable images; 39 (13%) were identified as having referable glaucoma. Sensitivity 91% and specificity 93%.
Rao et al. 2024 [7]	Tertiary Glaucoma Center, India (validation study)	Case (age > 18 years with glaucoma); Control (without glaucoma from general ophthalmology clinic); Exclusion: advanced cataract, corneal pathology, inflammation, neurological, shallow angle, postoperative subjects	Trained optometrist	Handheld smartphone fundus camera (42 degrees). Tabletop/desktop fundus camera (30 degrees)	No	Inbuilt AI algorithm (ResNet-50)	BCVA, slit lamp, IOP, gonioscopy, dilated fundus, SD-OCT, visual field examination	Sensitivity 93.7% and specificity 85.6% for referable glaucoma. AI detected 94% of glaucoma cases versus 60% by specialists grading the same images.
Senthil et al. 2025 [8]	Tertiary-care glaucoma clinic, India (validation study)	Aged 18 years and older with (Case) and without glaucoma (Control)	Experienced optometrist	Smartphone-based portable fundus camera (42 degrees)	Only if significant media opacity	Inbuilt offline AI algorithm	Refraction, BCVA, IOP, gonioscopy, slit lamp, indirect ophthalmoscopy. Additional investigation: VFA, SD-OCT for suspected and confirmed LOCS III	Accuracy 92.0%, sensitivity 91.4%, and specificity 94.1%. Sensitivity increased with glaucoma severity from 86.9% in early to 96.0% in advanced disease.
Jan et al. 2025 [10]	General practice clinics, Australia (primary-care implementation)	Aged 50 years and older attending a general practice clinic	Staff	Fixed fundus camera (50 degrees)	No	AI-based glaucoma detection	General health, previous ocular history	277/414 participants (66.9%) had analyzable images. AUROC 0.80, sensitivity 65.0%, and specificity 94.6%; 18/161 (11.2%) previously undiagnosed participants were identified as referable. 37.5% referred participants attended an eye-care provider.
Lima-Cabrita et al. 2026 [11]	Primary healthcare integrated with diabetic retinopathy screening, Portugal (screening pathway implementation)	Aged 55-65 years with and without diabetes. Exclusion: bilateral blindness or severe mobility limitation	Certified orthoptist	Fixed fundus camera	No	MONA-GLC AI system	IOP	671/1038 (65%) attended screening; AI referred 10% versus 18% by expert assessment. Sensitivity 78% and specificity 95%; ICER €1725/QALY at 1% prevalence.

The available studies vary in the implementation outcomes they report. While some report image gradability, referral yield, diagnostic performance, screening attendance, acceptability, or cost-effectiveness, downstream outcomes such as referral adherence, time to diagnosis, treatment initiation, and loss to follow-up remain insufficiently reported. These outcomes are important for determining whether AI-assisted screening translates into effective glaucoma care beyond initial detection.

In settings where access to specialist glaucoma care is limited, the relevant questions are not simply whether an algorithm can classify a fundus photograph, but whether the image can be acquired reliably at a vision center, whether the system can function with limited connectivity, whether non-specialist personnel can use it safely, and whether patients identified as potentially having glaucoma can be linked to appropriate confirmatory care [6-8,10,11]. These implementation considerations may ultimately determine the population-level value of AI-assisted screening more than incremental improvements in algorithmic accuracy.

An equally important consideration is the population and clinical environment in which AI systems are evaluated. The evidence remains heterogeneous, and performance reported in one setting may not apply directly to others. Studies from tertiary glaucoma centers have often relied on case-control populations with established glaucoma and selected controls, whereas studies conducted in primary care or community settings have generally included broader age-defined populations. This distinction is particularly relevant when interpreting AI performance for population screening. Many AI models have been developed or evaluated using datasets enriched with individuals who have established glaucoma and relatively healthy controls, often from tertiary or specialist-care settings. Such populations may facilitate algorithm development, but they do not necessarily reflect the clinical spectrum encountered in community screening, where disease prevalence is lower and individuals may have a wider range of optic-disc appearances, coexisting ocular conditions, image quality, and disease severity. This difference in spectrum can influence measures of diagnostic performance and, importantly, the positive predictive value of an AI-based screening test. Even a system with high sensitivity and specificity may generate many false-positive referrals when applied to a lower-prevalence population, potentially increasing the burden on already limited specialist services. Under-representation of population groups in training data may contribute to algorithmic bias. Differences in optic-disc morphology across ethnic populations and variation in disease severity or coexisting ocular conditions may also affect performance. Evaluation should report sensitivity, specificity, and image gradability across relevant demographic and clinical subgroups, where sample sizes permit. Changes in cameras, image acquisition, and clinical settings may introduce domain shift and should be assessed before deployment. Prospective evaluation across representative populations, subgroups, and real-world imaging environments is therefore needed to establish clinical utility and health-system impact.

Lessons from real-world deployments

Several practical lessons emerge from these studies. First, non-mydriatic imaging is becoming central to deployment. As demonstrated by the implementation studies discussed above, portable smartphone-based fundus cameras can facilitate non-mydriatic image acquisition at rural and satellite vision centers, potentially reducing the need for specialist ophthalmic imaging infrastructure at the point of screening [6]. Lima-Cabrita et al. evaluated AI-assisted glaucoma screening within an existing retinal screening pathway using non-mydriatic imaging [11]. This approach highlights how glaucoma assessment can be incorporated into an established screening workflow rather than requiring a separate ophthalmic service. By using existing imaging infrastructure and avoiding pharmacological dilation, screening can be incorporated with relatively limited additional requirements at the point of assessment. The key consideration, however, is whether individuals identified as being at increased risk can be appropriately referred for confirmatory glaucoma evaluation and subsequently linked to treatment when required.

Second, the choice of imaging platform is likely to depend on where screening is being delivered. Upadhyaya et al. and Rao et al. demonstrated the feasibility of handheld smartphone-based systems in India, whereas Lima-Cabrita et al. and Jan et al. used table-top fixed fundus cameras within established primary care or screening environments [6-8,10,11]. These studies suggest that no single imaging platform is optimal for all settings. Portability may be more useful for rural and peripheral centers, while fixed systems may be appropriate where dedicated screening infrastructure already exists. The relevant question here is therefore not which camera performs best in isolation, but which system can produce consistently gradable images within the workflow of the intended service.

Third, these studies support the potential for task-sharing. In several implementation studies, images were acquired by vision technicians, optometrists, or orthoptists rather than ophthalmologists [6,7,11]. This allows specialists to focus on assessing patients identified as potentially having glaucoma, rather than requiring an ophthalmologist to be present at every screening encounter. This may be important for extending screening beyond specialized centers.

Fourth, AI is more likely to be scalable when it is embedded within an existing care pathway rather than introduced as a standalone program. The Portuguese experience of incorporating glaucoma assessment into diabetic retinopathy screening is instructive [11]. Rather than creating a separate infrastructure for every disease, the same retinal image and screening encounter can potentially support assessment for multiple conditions. Such integration may be especially valuable in low- and middle-income countries (LMICs), where parallel disease-specific programs may be difficult to sustain.

Fifth, economic value becomes important once screening moves from feasibility to scale. The value of AI-based glaucoma screening depends not only on the cost of screening technology but also on disease prevalence and what happens after a positive result. In the Portuguese study, the estimated screening cost was EUR 11.65 per participant, with an incremental cost-effectiveness ratio (ICER) of EUR 1725 per quality-adjusted life year (QALY) at a glaucoma prevalence of 1%; screening became cost-saving at a prevalence of 2% or higher [11]. A Dutch model of repeated population-wide AI screening for primary open-angle glaucoma estimated that, compared with opportunistic case finding, screening could detect approximately 1.6 times more glaucoma cases and generate an additional 0.014 QALYs per invited person, with a societal perspective ICER of EUR 19,311 per QALY. The probability of cost-effectiveness was 51.2% at EUR 20,000 per QALY and 94.7% at EUR 50,000 per QALY [13]. Specificity influenced cost-effectiveness more than sensitivity because false-positive referrals generate additional confirmatory visits and downstream costs. Thus, the economic feasibility of AI in glaucoma screening depends on prevalence, screening costs, referral compliance, and the cost of false-positive referrals. These parameters are likely to differ substantially between health systems and therefore should not be assumed to be transferable across settings.

These experiences across Indian and other healthcare systems suggest that successful deployment is less about identifying a universally superior camera or algorithm and more about choosing technology that fits the practical realities of the health system. Portability, non-mydriatic imaging, task-sharing, offline functionality, integration with existing programs, and economic feasibility are interconnected components of implementation. For India, these considerations will become increasingly important as AI moves from individual pilot projects towards more structured primary eye-care delivery. The Strategy for Artificial Intelligence in Healthcare for India (SAHI) provides an important policy context for this transition, emphasizing the need for AI to be used safely, responsibly, and at a scale that can be sustained within existing health systems [14]. For glaucoma, the goal should therefore not be to introduce AI as another standalone screening tool but to integrate it thoughtfully into existing primary and secondary eye-care pathways. Its usefulness will ultimately depend on how well it supports clinical decision-making, appropriate referral, and timely access to specialist care.

From AI output to clinical care

The transition from algorithm validation to routine clinical deployment introduces challenges that are often not captured in controlled research settings. In routine practice, fundus image quality can vary considerably because of cataracts, small pupils, media opacity, poor fixation, or differences in image acquisition. Portable cameras can make screening more accessible, but they also increase the need for appropriate training and quality control. AI performance may also be affected by bias in training data, particularly when the populations, imaging devices, or disease spectrum differ from those represented in the development datasets. An algorithm that performs well with good-quality images may not perform in the same way when used with the wider range of images encountered in primary eye care.

It is also important to distinguish screening from diagnosis. In current implementation studies, an AI-generated result does not replace a comprehensive glaucoma assessment. Patients flagged as being at risk may still require intraocular pressure measurement, OCT, visual field testing, and examination by an ophthalmologist. A negative AI result should also be interpreted cautiously given potential biases in training data and differences between development and real-world populations. At present, AI's more realistic role is to support screening and help identify individuals who need further assessment, rather than to establish a diagnosis independently.

The bigger challenge may lie in what happens after the AI generates a result. Moving from AI development and validation to routine clinical adoption requires more than generating an accurate output; screening must be supported by personnel who can obtain a good-quality image, a reliable system to record and communicate results, access to appropriate confirmatory investigation, and a clear pathway for follow-up and treatment. As these systems become more integrated into routine care, other issues, including integration with health records and referral systems, regulatory oversight, data governance, cybersecurity, and ongoing monitoring of performance, will also need attention.

The success of an AI-based screening system should therefore not be judged by classification accuracy alone. An accurate algorithm has limited practical value if there is no clear, practical pathway for managing the people it identifies. Conversely, a system with somewhat lower performance under controlled conditions may have greater public health value in practice if it is reliable with real-world images, can operate in settings with limited connectivity, is simple enough for non-specialist personnel to use, and can be integrated into an existing eye-care network. The emphasis should therefore be on how well the entire screening pathway works, rather than on algorithm performance in isolation.

From technology to standards

Indian studies have identified practical requirements for AI-assisted diabetic retinopathy (DR) screening, including local validation, integration with available cameras and staff, reliable power supply, technical support, and measures to improve screening uptake [15,16]. These findings may inform the planning of AI-assisted glaucoma screening, although they do not establish its effectiveness. The All India Ophthalmological Society (AIOS), in collaboration with the Vitreoretinal Society of India (VRSI), has developed guidance addressing screening eligibility, referral pathways, quality assurance, training, governance, and the potential role of AI in image grading [17]. A similar consensus-based framework for AI-assisted glaucoma screening is needed, particularly because an AI-generated glaucoma suspicion is not a diagnosis. Individuals identified through screening may require IOP measurement, optic nerve evaluation, OCT, visual field testing, gonioscopy, and clinical examination before treatment decisions. Guidance should therefore define how positive, negative, and ungradable results are interpreted and linked to confirmatory care.

Quality control should be an integral part of such a framework. AI performance can change when systems are used across cameras, populations, image qualities, and clinical settings, and validation performance may not fully reflect routine practice. A quality-assurance process could include monitoring image quality and ungradable rates, periodically auditing AI outputs, and independently assessing a representative subsample of AI-negative cases to identify potential false-negative results. Where an AI system provides a probability or risk score, calibration should be assessed in the intended screening population. Referral thresholds selected in tertiary-care populations should not be assumed to be suitable for community screening. Local evaluation should assess sensitivity, specificity, positive predictive value, and referral workload before adopting a threshold. Ongoing monitoring is also important to detect performance drift following changes in camera, patient populations, workflows, or software versions. Experience from AI-assisted DR screening in India also supports periodic assessment after deployment, including monitoring of diagnostic performance, image gradability, and referral recommendations within routine services [18].

The AIOS, together with the Glaucoma Society of India and other relevant stakeholders, could consider developing minimum standards for image acquisition, quality assurance, referral thresholds, confirmatory assessment, documentation, follow-up, and ongoing monitoring. Such a framework would also support consistent evaluation of implementation outcomes, including referral yield, referral adherence, time to diagnosis, treatment initiation, loss to follow-up, and cost-effectiveness (Table 2). The focus should therefore extend beyond algorithmic accuracy to whether AI-assisted screening facilitates timely and appropriate glaucoma care.

**Table 2:** Implementation outcomes for AI-assisted glaucoma screening

Outcome	Definition and relevance
Referral yield	Proportion of screened individuals identified as requiring further glaucoma assessment; indicates the referral workload generated by screening.
Referral adherence	Proportion of all referred individuals who complete the recommended assessment within a defined period; directly affects screening effectiveness.
Time to diagnosis	Time from a positive screening result to a definitive diagnostic decision; indicates delays in diagnostic assessment.
Treatment initiation	Proportion of individuals for whom treatment is recommended who receive the indicated treatment within a defined period; measures linkage to treatment.
Loss to follow-up	Proportion without documented completion of the next required care step within a defined period; reported separately for referral, diagnostic assessment, and treatment follow-up.
Cost-effectiveness	Assesses whether the resources required for AI-assisted screening are justified by the health outcomes achieved.

The conceptual framework illustrates the proposed pathway from population selection and image acquisition at the community or primary-care level through AI-assisted assessment, human oversight, referral, confirmatory assessment, diagnosis, treatment, and follow-up (Figure 1). It also highlights priority evidence gaps relevant to implementation, including image quality, risk stratification, referral pathways, and monitoring of AI performance.

**Figure 1:**
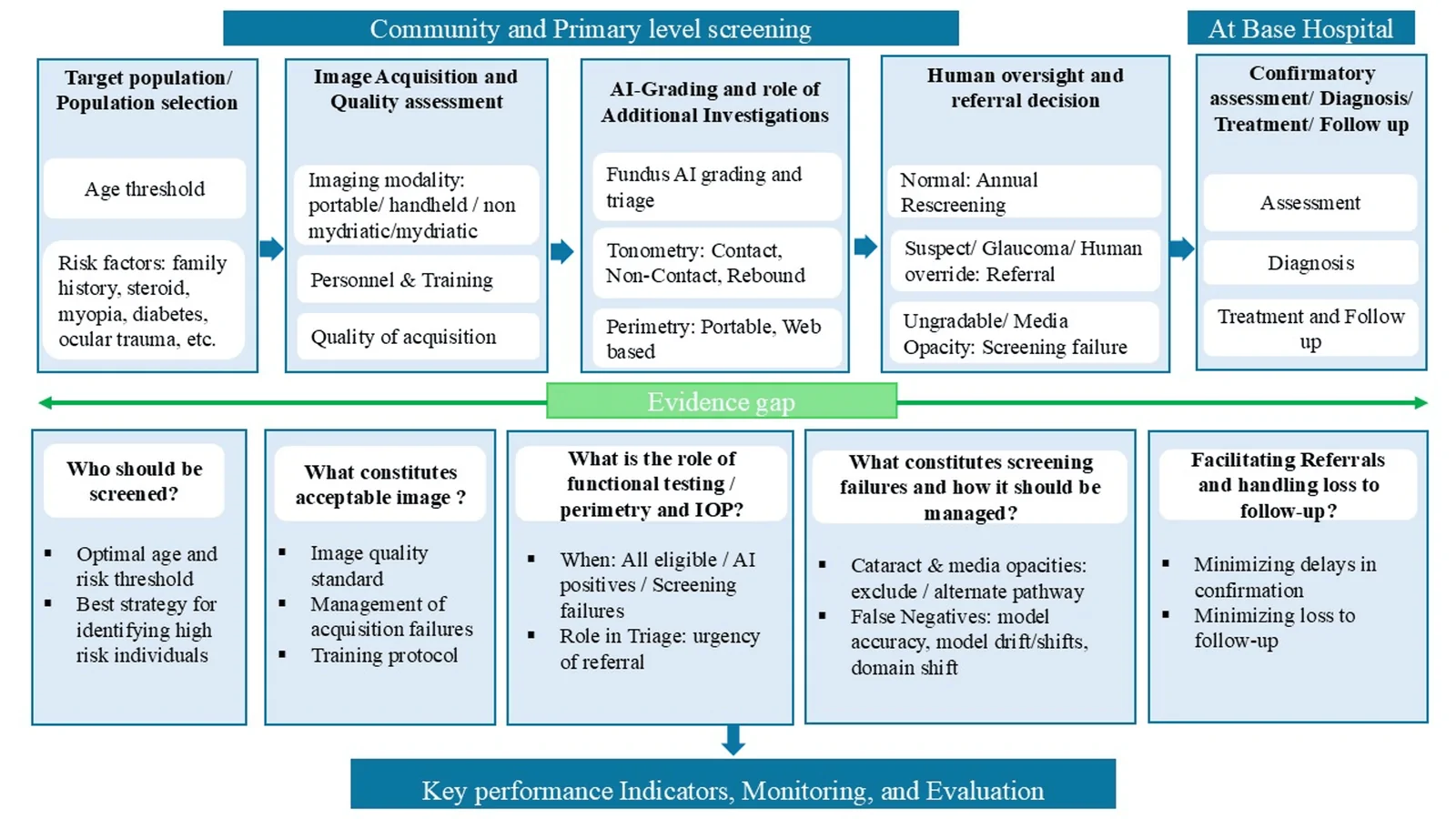
Proposed conceptual framework for AI-assisted glaucoma screening across the continuum of care Conceptual framework developed by the authors from the reviewed literature; it is not a validated clinical protocol. The figure was created using Microsoft PowerPoint (Microsoft Corporation, Redmond, WA, USA).

Beyond detection: building a pathway to care

The future of glaucoma screening is likely to depend not only on improving algorithm performance but also on developing screening pathways that are practical for routine primary eye care. The clinical value of AI-assisted screening ultimately depends on what happens after an individual is identified as being at increased risk. Timely access to confirmatory assessment and subsequent management is therefore essential to ensure that screening translates into meaningful patient care. This requires clearly defined referral pathways, adequate capacity at receiving facilities, and systems to support follow-up and minimize loss to care.

In the Australian primary-care study, only 37.5% of participants advised to seek further assessment reported attending an eye-care provider [10]. In a non-AI-targeted glaucoma-screening program in Odisha, 152 of 261 individuals referred from community settings attended further examination (58.2%), compared with 1,880 of 1,958 referred within the base hospital (96.0%) [19]. Referral support may help improve attendance. In the NYC-SIGHT cluster-randomized trial, attendance at the initial in-office eye examination was 51.8% with patient navigation compared with 38.1% under usual care [20]. These findings highlight the importance of evaluating linkage to care separately for community and facility-based screening and the need to identify mechanisms to improve referral adherence.

Media opacity is one practical challenge that deserves greater attention. Dense cataract and other media opacities can limit fundus image quality and, in some cases, make the image impossible to interpret. Recognizing media opacity as part of the screening process, rather than simply a technical limitation of imaging, may improve the efficiency of community-based glaucoma screening. Patients with visually significant cataracts that prevent adequate fundus imaging should enter an alternative clinical assessment pathway. Cataract and suspected glaucoma should be assessed together, with glaucoma investigations undertaken as clinically indicated and feasible. These patients should not be classified as screen-negative for glaucoma. This reframes an ungradable image from a technical limitation of the screening system to a clinically relevant finding requiring an appropriate alternative pathway. Screening algorithms and referral protocols should therefore distinguish between a gradable negative image, an ungradable image, and a positive finding requiring further evaluation, rather than treating all ungradable images as evidence of suspected glaucoma. Such an approach may improve the efficiency of community-based screening while ensuring that patients with potentially treatable media opacity are not lost within an AI-driven referral pathway.

A second opportunity is to bring functional assessment closer to primary eye care. Most image-based approaches identify structural features suggestive of glaucoma, whereas functional loss remains central to the disease. Portable and tablet-based perimetry could potentially complement structural assessment in settings where conventional automated perimetry is not readily available, but should not be considered a substitute for standard automated perimetry when the latter is available. An Indian study comparing tablet perimetry with the Humphrey field analyzer demonstrated the feasibility of this approach, although its performance did not support replacing conventional standard automated perimetry for glaucoma screening [21]. Similar findings were reported by Richardson et al. [22]. The evidence related to portable glaucoma technologies remains largely heterogeneous. A recent scoping review of 216 studies found that the large majority were method agreement studies (90.3%), with relatively limited evidence on their effectiveness when incorporated into routine care pathways [23]. The potential value of portable perimetry within an AI-assisted screening pathway lies in complementing structural assessment and helping determine which individuals require further specialist evaluation. Thus, portable perimetry may have a complementary role, but current evidence is insufficient to recommend its routine inclusion in AI-assisted glaucoma screening. Further evidence from real-world primary and community settings is needed to establish how portable functional testing can be integrated into screening, referral and follow-up pathways.

These considerations suggest a layered approach to community-based glaucoma screening that requires prospective evaluation and consensus. Known risk factors, such as a family history of glaucoma, diabetes, and prolonged corticosteroid use, may help identify individuals for targeted screening. However, the optimal combination of risk factors and eligibility criteria for AI-assisted screening in community and primary-care settings remains to be established. AI analysis of fundus photographs could identify optic-disc features suspicious for glaucoma and help select individuals for further clinical assessment. Visual field testing could provide additional functional information where feasible, although the role of portable perimetry within community screening pathways requires further evaluation. Such approaches may be particularly relevant in settings where access to conventional diagnostic equipment and specialist services is limited.

The larger challenge, however, is to ensure that screening remains connected to care. A technically successful screening program may have limited value if positive findings cannot be translated into timely diagnostic evaluation and treatment [24]. In this context, AI is best seen as one part of the screening pathway, rather than the pathway itself. Its usefulness will depend on how effectively it integrates into the existing primary eye-care pathway and links to appropriate referral and follow-up systems. A useful system should make screening easier for both patients and health care providers, rather than creating additional steps or complexity. In India, the focus should therefore be on using AI where it adds clear value and integrating it into simple, stepwise screening pathways that can support early detection and facilitate timely referral for specialist evaluation and care.

## Conclusions

AI-assisted glaucoma screening can extend glaucoma assessment into primary and community eye-care settings, particularly through portable imaging, task-sharing, and integration with existing screening services. However, algorithmic performance alone does not establish the effectiveness of a screening program. The value of AI depends on reliable image acquisition, appropriate patient selection, recognition and management of ungradable images, human oversight, effective referral pathways, and access to confirmatory diagnosis and treatment. Future implementation should therefore focus on integrating AI into practical, stepwise screening pathways rather than using it as a standalone diagnostic tool. Consensus-based standards addressing image quality, quality assurance, referral, confirmatory assessment, documentation, and monitoring of real-world outcomes will be important as these technologies move towards routine clinical use. Ultimately, AI-assisted glaucoma screening should be evaluated across the continuum of care from identifying individuals at risk to ensuring timely diagnosis, treatment, and follow-up.
